# Characteristics and Outcomes among Older HIV-Positive Adults Enrolled in HIV Programs in Four Sub-Saharan African Countries

**DOI:** 10.1371/journal.pone.0103864

**Published:** 2014-07-30

**Authors:** Eduard Eduardo, Matthew R. Lamb, Sasi Kandula, Andrea Howard, Veronicah Mugisha, Davies Kimanga, Bonita Kilama, Wafaa El-Sadr, Batya Elul

**Affiliations:** 1 Department of Epidemiology, Mailman School of Public Health, Columbia University, New York, New York, United States of America; 2 ICAP, Mailman School of Public Health, Columbia University, New York, New York, United States of America; 3 National AIDS and STI Control Programme, Ministry of Health, Nairobi, Kenya; 4 National AIDS Control Program, Ministry of Health and Social Welfare, Dar es Salaam, Tanzania; UCL Institute of Child Health, University College London, United Kingdom

## Abstract

**Background:**

Limited information exists on adults ≥50 years receiving HIV care in sub-Saharan Africa.

**Methodology:**

Using routinely-collected longitudinal patient-level data among 391,111 adults ≥15 years enrolling in HIV care from January 2005–December 2010 and 184,689 initiating ART, we compared characteristics and outcomes between older (≥50 years) and younger adults at 199 clinics in Kenya, Mozambique, Rwanda, and Tanzania. We calculated proportions over time of newly enrolled and active adults receiving HIV care and initiating ART who were ≥50 years; cumulative incidence of loss to follow-up (LTF) and recorded death one year after enrollment and ART initiation, and CD4+ response following ART initiation.

**Findings:**

From 2005–2010, the percentage of adults ≥50 years newly enrolled in HIV care remained stable at 10%, while the percentage of adults ≥50 years newly initiating ART (10% [2005]-12% [2010]), active in follow-up (10% [2005]-14% (2010]), and active on ART (10% [2005]-16% [2010]) significantly increased. One year after enrollment, older patients had significantly lower incidence of LTF (33.1% vs. 32.6%[40–49 years], 40.5%[25–39 years], and 56.3%[15–24 years]; p-value<0.0001), but significantly higher incidence of recorded death (6.0% vs. 5.0% [40–49 years], 4.1% [25–39 years], and 2.8% [15–24 years]; p-valve<0.0001). LTF was lower after vs. before ART initiation for all ages, with older adults experiencing less LTF than younger adults. Among 85,763 ART patients with baseline and follow-up CD4+ counts, adjusted average 12-month CD4+ response for older adults was 20.6 cells/mm^3^ lower than for adults 25–39 years of age (95% CI: 17.1–24.1).

**Conclusions:**

The proportion of patients who are ≥50 years has increased over time and been driven by aging of the existing patient population. Older patients experienced less LTF, higher recorded mortality and less robust CD4+ response after ART initiation. Increased programmatic attention on older adults receiving HIV care in sub-Saharan Africa is warranted.

## Introduction

Recent UNAIDS and WHO data on the HIV epidemic show a considerable increase in the number of people receiving antiretroviral therapy (ART), and sharp declines in both the number of new infections and HIV-related deaths [1], [2]. Consequently, the number of people living with HIV (PLWH) [1], [2] and their life expectancy have increased to their highest levels [3]. These accomplishments bring hope in the fight against the epidemic but also new challenges.

Worldwide trends suggest a “graying” of the HIV epidemic with an increasing proportion of PLWH aged 50 years or older. In the United States, the proportion of older PLWH increased from 17% in 2001 to 24% in 2005 [4], and is projected to rise to 50% by 2015 [5]. This demographic shift is not limited to resource-rich settings. In 2011, 3.1 million adults 50 years or older were estimated to be living with HIV in sub-Saharan Africa, accounting for 13% of all infections in the region [6]. Given the aforementioned reduction in HIV-related mortality due to expansion of ART use [1], [2], coupled with the occurrence of secondary incidence peaks of HIV acquisition among older adults in sub-Saharan Africa [7], the number of older PLWH will likely continue to increase in this region [6], [8]–[10]. Despite this, information on what accounts for this shift and the characteristics of and outcomes among older PLWH in sub-Saharan Africa remains scant [8], [11]–[13].

Older PLWH face unique challenges that may hinder timely and effective HIV care and treatment. Symptoms of advanced HIV disease (e.g. fatigue, weight loss, cognitive deficits) resemble those associated with aging and therefore may result in delayed HIV diagnosis, enrollment in care and initiation of treatment, a common occurrence in sub-Saharan Africa and a leading cause of HIV-related death [2], [8], [14], [15]. Chronic conditions (e.g. hypertension, diabetes) associated with aging require treatment with medications, augmenting the likelihood of drug-drug interactions with ART and adverse drug reactions [16]–[18]. Furthermore, immune recovery decreases with age, particularly for HIV-positive individuals, as thymus function diminishes leading to increased risk of immunologic failure and death [19]–[21]. Lastly, in sub-Saharan Africa, older PLWH often face the financial and psychosocial responsibility of raising grandchildren orphaned by HIV [22], [23], which may compromise engagement and retention in HIV care.

We used routinely-collected patient-level data from a large, multi-country HIV program in sub-Saharan Africa to: (1) estimate the proportion of all newly enrolled and active adult patients receiving HIV care and initiating ART over time who were ≥50 years, and (2) compare key baseline characteristics and outcomes between PLWH ≥50 years and <50 years of age.

## Materials and Methods

### Study Population

The study population included patients ≥15 years of age enrolled into HIV care between January 01, 2005 and December 31, 2010 and followed through December 31, 2011 at 199 health facilities supported by ICAP at Columbia University through funding from the President's Emergency Plan for AIDS Relief (PEPFAR). These included 69 facilities in Kenya, 52 in Tanzania, 44 in Rwanda and 34 in Mozambique. Provision of services at each clinic was governed by respective national guidelines.

### Data Sources

Patient information routinely collected at each clinic visit was documented by clinicians on national patient forms, and regularly entered into on-site electronic databases by data clerks. Information was collected at enrollment and subsequent follow-up visits, with visit frequency depending on patient CD4+ count and whether they were receiving ART. Data quality assessments were done at least annually at each health facility. Each quarter, these electronic data were de-identified, encrypted, and imported into a common-format database for analyses. Use of these de-identified patient-level data for research purposes was approved by the Columbia University Medical Center IRB and ethics committees in each of the four countries where patients were enrolled.

### Outcome definitions

The proportion of newly enrolled patients into HIV care and ART initiation who were ≥50 years were calculated for each quarter from 2005–2010. Patients were considered “active in care” if they were not documented as dead, transferred, or lost to follow-up (LTF) and if they had a recorded visit during the quarter (ART patients) or during the current or prior three quarters (pre-ART patients). Patients were followed until death, loss to follow-up, transfer, or December 31, 2011, whichever occurred first. Patients were considered LTF if they were not recorded as dead or transferred and did not have a recorded visit date for six months for ART patients or 12 months for pre-ART patients with no subsequent recorded visit. ART patients LTF were censored 15 days after their last recorded visit and pre-ART patients were censored 3 months after their last recorded visit. Patients with a documented transfer to another facility were censored at their transfer date.

Measures of HIV disease status (CD4+ cell count and WHO stage) at enrollment into HIV care and ART initiation were determined by assigning the closest recorded measure taken within three months prior to or one month after enrollment or ART initiation, as appropriate.

### Statistical Analysis

Trends over time in the proportion of patients active in HIV care or on ART were conducted after calculating each active patient's age at the beginning of a given quarter. For all other analyses, patients were classified into four age groups (15–24, 25–39, 40–49, and ≥50 years of age) based on their age at the time of enrollment into care or ART initiation.

We assessed trends over time in the proportion of all adults who were ≥50 years among the following groups using unadjusted log-binominal regression: (1) patients newly enrolling in HIV care, (2) patients newly initiating ART, (3) patients currently active in HIV care, and (4) patients currently on ART.

Baseline characteristics and outcomes (LTF and recorded death) through one year after enrollment into HIV care and ART initiation were compared between age groups using generalized linear mixed models and survival analytic techniques (Kaplan-Meier and Cox proportional models with robust sandwich estimators of variance), respectively. Comparisons of CD4+ cell count at enrollment into care and ART initiation were performed after log-transformation. CD4+ cell count response after ART initiation was compared between age groups for the subset of patients with a recorded CD4+ cell count at ART initiation and at least one follow-up CD4+ cell count. Change in CD4+ cell count after ART initiation was plotted as the median increase in CD4+ cell count by time after ART initiation, lagging each patient's recorded CD4+ count for up to three months or until the patient had another CD4+ cell count or was censored from the analysis. Statistical tests for differences in absolute overall change in CD4+ cell count after ART initiation by age group were conducted using repeated-measures linear regression, adjusting for country, sex, CD4+ count at ART initiation, year of ART initiation, facility type and location.

IRB and ethical board approval for use of this routinely-collected de-identified patient-level data has been given by the Columbia University Medical Center IRB, the Kenya Medical Research Institute Ethics Review Committee, the Comité Nacional de Bioética para Saúde (Mozambican National Ethic Committee), the Comite National de Pilotage des Recherches dans le Domaine du VIH/SIDA in Rwanda (National Steering Committee for HIV/AIDS Research in Rwanda), and the National Institute for Medical Research in Tanzania. This study involved the use of existing, routinely-collected de-identified patient-level data and consequently was given a non-research designation by the Columbia University Medical Center IRB and country ethical review boards. No interviews were conducted, and no data was collected independently for this study. Consequently, informed consent was not necessary or obtained.

## Results

**Figure 1:**
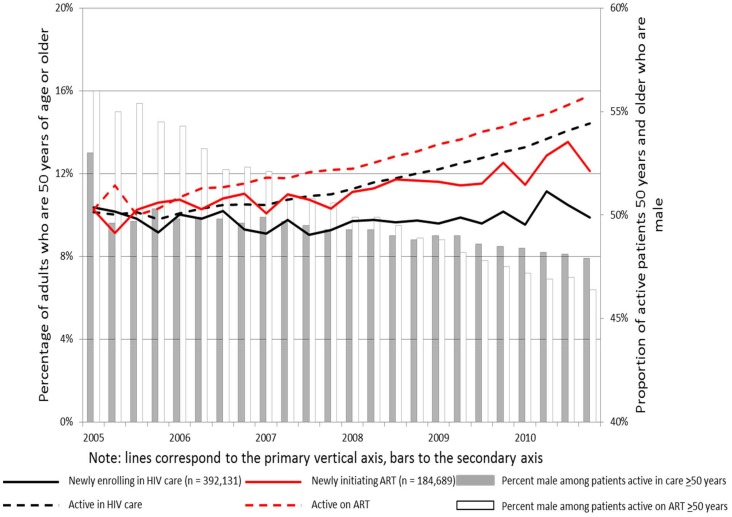
Proportion of adult patients 50 years and older, and proportion male, over time.

Over the six-year period of observation, 392,131 patients ≥15 years of age enrolled in HIV care (Table 1), 184,689 (47%) of whom initiated ART across the 199 health facilities (Table 2). 10% (N = 38,337) of the patients enrolling in HIV care and 11% (N = 21,108) of those initiating ART were ≥50 years of age. The percentage of patients newly enrolling into HIV care who were ≥50 years of age remained stable over time at around 10% (P = 0.33), while the percentage of patients remaining in HIV care who were ≥50 years increased from 10% to 14% over follow-up period (P<0.0001). The percentage of patients newly initiating ART who were ≥50 years increased from 10% to 12% (P<0.0001) over the study period, and the percentage of patients remaining active on ART who were ≥50 years increased from 10% to 16% (P<0.0001) (Figure 1).

**Table 1:** Characteristics of adult patients enrolling in HIV care by age group, 2005–2010.

		Overall		≥50 years		40–49 years		25–39 years		15–24 years	
		N	%	N	%	N	%	N	%	N	%
		392,131 (100%)		38,337 (10%)		77,457 (20%)		211,842 (54%)		64,495 (16%)	
**Country**	Kenya	87,992	22%	10,427	27%	19,980	26%	48,453	23%	9,132	14%
	Mozambique	211,849	54%	18,640	49%	36,797	48%	112,392	53%	44,020	68%
	Rwanda	38,038	10%	3,642	9%	8,582	11%	20,634	10%	5,180	8%
	Tanzania	54,252	14%	5,628	15%	12,098	16%	30,363	14%	6,163	10%
**Facility location**	Urban	197,588	50%	18,080	47%	37,160	48%	106,225	50%	36,123	56%
	Semi-Urban	109,037	28%	11,571	30%	23,434	30%	61,059	29%	12,973	20%
	Rural	85,506	22%	8,686	23%	16,863	22%	44,558	21%	15,399	24%
**Facility type**	Primary	139,245	36%	12,668	33%	25,500	33%	74,565	35%	26,512	41%
	Secondary/Tertiary	252,886	64%	25,669	67%	51,957	67%	137,277	65%	37,983	59%
**Enrollment year**	2005	35,404	9%	3,468	9%	8,422	11%	19,173	9%	4,341	7%
	2006	59,210	15%	5,820	15%	12,654	16%	32,117	15%	8,619	13%
	2007	74,738	19%	6,951	18%	14,409	19%	40,580	19%	12,798	20%
	2008	78,454	20%	7,622	20%	14,934	19%	42,453	20%	13,445	21%
	2009	75,526	19%	7,400	19%	14,129	18%	40,976	19%	13,021	20%
	2010	68,799	18%	7,076	18%	12,909	17%	36,543	17%	12,271	19%
**Point of Entry**	PMTCT	36,685	9%	311	0.8%	1,653	2%	21,053	10%	13,668	21%
	TB/HIV	9,419	2%	1,212	3%	2,340	3%	5,045	2%	822	1%
	VCT	147,340	38%	14,769	39%	30,954	40%	79,508	38%	22,109	34%
	Inpatient	20,307	5%	2,409	6%	4,195	5%	10,663	5%	3,040	5%
	Outpatient	27,048	7%	3,343	9%	6,057	8%	14,238	7%	3,410	5%
	Other*	117,510	30%	12,820	33%	25,348	33%	62,991	30%	16,351	25%
	Unknown	33,822	9%	3,473	9%	6,910	9%	18,344	9%	5,095	8%
**Sex**	Male	130,928	33%	19,058	50%	34,945	45%	66,942	32%	9,983	15%
	Female	261,203	67%	19,279	50%	42,512	55%	144,900	68%	54,512	85%
**Median (IQR) CD4+ Cell Count (% missing)**		259 (117–460) (49%)		224 (109–400) (47%)		216 (97–394) (46%)		256 (113–454) (49%)		377 (199–593) (55%)	
**N (%) WHO stage III/IV (% missing)**		132,752 (47%) (28%)		15,499 (53%) (24%)		30,553 (53%) (25%)		71,225 (47%) (28%)		15,475 (37%) (35%)	
**On TB Treatment at enrollment**		12,952	3%	1,459	4%	3,019	4%	7,255	3%	1,219	2%

*“Other” category includes mostly transfers from other facilities.

**Table 2:** Characteristics of adult patients initiating ART by age group, 2005–2010.

		Overall		≥50 years		40–49 years		25–39 years		15–24 years	
		N	%	N	%	N	%	N	%	N	%
Patients initiating ART		184,689		21,108 (11%)		43,955 (24%)		101,682 (55%)		17,944 (10%)	
**Year of ART initiation**											
2005		10,822	6%	1,097	5%	3,107	7%	5,894	6%	724	4%
2006		24,789	13%	2,663	13%	6,325	14%	13,763	14%	2,038	11%
2007		33,981	18%	3,585	17%	7,823	18%	18,976	19%	3,597	20%
2008		36,025	20%	4,121	20%	8,236	19%	19,997	20%	3,671	20%
2009		36,510	20%	4,291	20%	8,429	19%	20,091	20%	3,699	21%
2010		35,219	19%	4,406	21%	8,379	19%	18,950	19%	3,484	19%
2011*		7,343	4%	945	4%	1,656	4%	4,011	4%	731	4%
**Sex**											
Male		65,188	35%	10,405	49%	19,646	45%	32,037	32%	3,100	17%
Female		119,501	65%	10,703	51%	24,309	55%	69,645	68%	14,844	83%
**Median (IQR) CD4 cell count at ART initiation, cells/mm3**											
Overall	CD4 count	161 (77–243)		166 (90–245)		154 (75–234)		159 (74–242)		182 (89–272)	
	% missing	33%		33%		32%		33%		37%	
Males	CD4 count	144 (63–229)		152 (78–234)		140 (62–223)		141 (59–230)		159 (62–249)	
	% missing	32%		32%		31%		32%		36%	
Females	CD4 count	170 (86–250)		177 (103–256)		165 (88–242)		166 (82–247)		187 (95–276)	
	% missing	32%		34%		34%		34%		37%	
**WHO stage III/IV at ART initiation**											
Overall	% WHO III/IV	66,672 64%		8,166 66%		16,660 66%		36,128 63%		5,718 60%	
	% missing	43%		41%		42%		43%		47%	
Males	% WHO III/IV	25,762 70%		4,089 67%		7,774 70%		12,752 71%		1,147 70%	
	% missing	43%		41%		43%		44%		47%	
Females	% WHO III/IV	40,910 60%		4,077 62%		8,886 61%		23,376 58%		4,571 56%	
	% missing	43%		40%		42%		43%		47%	
**Documented tuberculosis treatment at ART initiation**		4,522 (2%)		470 (2%)		1,149 (3%)		2,580 (3%)		323 2(%)	

*ART initiation in 2011 was through June 30^th^ only for those enrolling in care by the end of 2010.

Fifty percent of patients aged ≥50 were male compared with 45%, 32% and 15%, of those aged 40–49 years, 25–39 years, and 15–24 years, respectively. The proportion of patients aged ≥50 active in HIV care and on ART who were male decreased over time (P<0.0001 for trend) (Figure 1). CD4+ cell counts at enrollment into care and ART initiation were available for 51% and 67% of all patients, respectively (Tables 1 and 2). At enrollment into HIV care, median CD4+ cell count among older patients was 224 cells/mm^3^ (IQR: 109–400) and was similar to those aged 40–49 years (216 cells/mm^3^ [97–394]) but lower than those aged 25–39 years (256 cells/mm^3^ [113–454]) and 15–24 years (377 cells/mm^3^ [199–593]) (P<0.0001 for overall difference in medians) (Table 1). At ART initiation (Table 2), older patients had slightly higher median CD4+ cell count (166 cells/mm^3^ [90–245]) compared with adults aged 40–49 years (154 cells/mm^3^ [75–234]) and those aged 25–39 years (159 cells/mm^3^ [74–242]), but significantly lower than those aged 15–24 years (182 cells/mm^3^ [89–272]) (P<0.0001). Information on WHO stage was available at enrollment into care and ART initiation for 72% and 57% of all patients, respectively. Nearly two-thirds of patients with WHO stage initiated treatment at WHO stage III or IV, with no substantial differences between age groups. For each age stratum, male patients initiated treatment at more advanced stages of HIV disease as measured by CD4+ cell count. The proportion of patients on treatment for tuberculosis at enrollment and at ART initiation was similar across all age strata (Tables 1 and 2).

Half of the patients received HIV care in urban areas, and 64% attended secondary or tertiary facilities, with little substantive difference by age group. Point of entry into HIV care was similar across age groups with the exception of those from prevention of mother to child transmission (PMTCT) programs. Less than 1% of patients ≥50 years of age were referred from PMTCT settings, compared with 2%, 10%, and 21% of those aged 40–49, 25–39 and 15–24 years (Table 1).

Table 3 shows the cumulative incidence of LTF and recorded death through one year after enrollment into HIV care and ART initiation by age group. Cumulative incidence of LTF through one year after enrollment into HIV care among patients ≥50 years of age was similar (33.1%) to that observed among patients 40–49 years of age (32.6%), but significantly lower than that of younger age groups (40.5% for adults 25–39 years and 56.3% for adults 15–24 years; P<0.0001 for difference between groups). Incidence of recorded death was significantly higher among older patients (6.0%) versus 5.0% for 40–49 years, 4.1% for 25–39 years, and 2.8% for 15–24 years (P<0.0001 for difference between groups). Across all age groups, men experienced higher incidence of LTF and death compared with women.

**Table 3:** Loss to follow-up and death one year after enrollment into care and ART initiation, by age group.

	Cumulative incidence* of loss to follow-up and recorded death one year after enrollment into HIV care** n = 392,131					
	% Loss to follow-up			% Recorded Dead		
Age Group (years)	Overall	Males	Females	Overall	Males	Females
≥50	33.1	35.9	30.5	6.0	7.3	4.7
40–49	32.6	36.5	29.4	5.0	6.5	3.9
25–39	40.5	46.1	38.0	4.1	5.9	3.3
15–24	56.3	58.5	55.9	2.8	4.3	2.5

	Cumulative incidence* of loss to follow-up and recorded death one year after ART initiation** n = 184,689					
	% Loss to follow-up			% Recorded Dead		
Age Group (years)	Overall	Males	Females	Overall	Males	Females
≥50	16.1	17.8	14.5	6.0	7.1	4.8
40–49	15.3	17.5	13.6	5.1	6.4	4.1
25–39	19.3	24.1	17.2	4.5	6.6	3.6
15–24	28.0	32.9	26.9	4.4	5.6	4.1

*Cumulative incidence of loss to follow-up and death estimated using Kaplan-Meier survival analytic techniques.

**Log-rank test for equality over strata statistically significant for all models at an alpha of 0.05.

LTF after ART initiation was lower than after enrollment into HIV care for all age and sex strata. Among patients who initiated ART, cumulative incidence of LTF was similar among adults ≥50 years and those 40–49 years of age (16.1% and 15.3%, respectively) but significantly lower than among younger age groups (19.3% for adults 25–39 years and 28.0% for adults 15–24 years); P<0.0001 for difference between groups. Recorded death through one year after ART initiation was highest among the oldest age group (6.0%) compared with adults 40–49 years (5.1%), 25–39 years (4.5%), and 15–24 years (4.4%); P<0.0001 for difference between groups. For each age-category, male patients were more likely to be LTF and to have died within a year after enrollment or ART initiation (Table 3).

**Figure 2:**
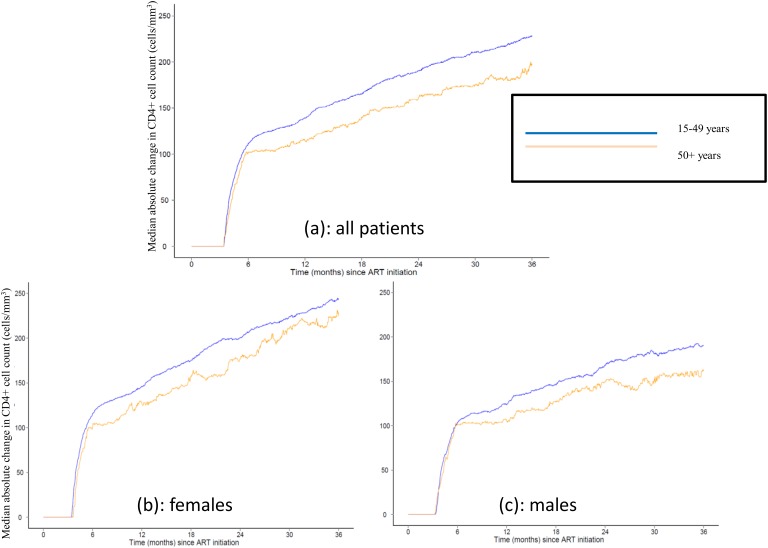
Change in CD4+ cell count after ART initiation, by age category. **2a.** All Patients. **2b.** Males. **2c.** Females.

Figure 2 presents CD4+ cell count response after ART initiation for the 85,763 (46%) patients with CD4+ cell counts at ART initiation and at least one follow-up CD4+ cell count. Among these patients, older adults experienced smaller increases in CD4+ cell count after ART initiation than all other age groups. This result was observed overall and when stratified by gender. After adjustment for baseline CD4+ cell count at ART initiation, sex, year of ART initiation, facility type, location, and country, compared to adults 25–39 years of age, the average CD4+ cell increase at 12 months among adults ≥50 years was 20.6 cells/mm^3^ lower (95% CI: 17.1 to 24.1 cells/mm^3^) (Table 4). The full results of the regression results are in Table S1.

**Table 4:** Multivariate regression: Relative increase in CD4+ cell count (cells/mm^3^) after ART initiation, n = 85,763.

	Model1^1^		Model2^2^	
Age Group	Change in CD4	95% CI	Change in CD4	95% CI
≥50 years	−33.9	(−30.2 to −37.6)	−20.6	(−17.1 to −24.1)
40–49 years	−12.7	(−9.8 to −15.6)	−8	(−5.2 to −10.7)
25–39 years	Reference			
15–24 years	1.3	(−4.3 to 6.8)	5.5	(0.4 to 10.5)

1 Controls for timing of CD4 measure after ART initiation and within-clinic correlation.

2 Additionally controls for clinic type and location, country, sex, year of ART initiation, and CD4 at ART initiation.

## Discussion

Our study—which reports on the largest number of PLWH ≥50 years of age enrolled in HIV programs in sub-Saharan Africa to date—showed that the percentage of older adults newly initiating ART and active in HIV care or on ART increased significantly from 2006 to 2010, while the percentage of such patients newly enrolling in HIV care remained stable during that period. These findings suggest that the observed increases in the percentage of patients aged ≥50 years initiating ART, and remaining active in care is resulting from a “graying” of the existing patient population rather than new enrollment of older patients. We found that approximately 14% and 16% of adult patients active in HIV care and on ART, respectively, were ≥50 years of age at the end of 2010, similar to population estimates of the percentage of older PLWH in sub-Saharan Africa [24]. Furthermore, in our analyses, 12% of the adult population newly initiating ART were ≥50 years of age at the end of 2010, similar to estimates from a recent study which included data from nine countries in sub-Saharan Africa [25].

We observed that patients ≥50 years of age enrolling into HIV care and initiating ART were more likely to be male compared to younger adults, which were disproportionately female. Our findings of disproportionately higher proportions of males among older adults compared to younger adults accessing HIV care is consistent with findings from a retrospective cohort study in Zomba District, Malawi [26], which compared adults 25–49 years of age at ART initiation to those 50 years and older, and the DART clinical trial in Zimbabwe and Uganda [27], which compared symptomatic (WHO stage 2–4) adults at ART initiation with CD4+ cell counts <200 cells/mm^3^ 18–49 years of age to those 50 years and older. We extend these studies by additionally presenting higher proportions of older adults enrolling in HIV care who are male, in addition to the findings at ART initiation, and by further stratifying into age categories of 15–24 years, 25–39 years and 40–49 years to highlight that the proportion male increases with each increasing age category.

The predominance of active males among patients ≥50 years in HIV care or on ART remained even though males were more likely to die or become LTF. This gender differential may reflect a higher prevalence of HIV among males ≥50 years, consistent with epidemiologic surveys in South Africa [28], and/or delayed engagement in care among men. As shown in our analyses and the published literature, male patients in resource-limited settings present for care at more advanced stages of HIV disease and therefore are more likely to initiate treatment late [29]–[31].

The imbalance in gender distribution by age does not completely explain the disparity in mortality between older and younger adults. Age-stratified analyses showed that mortality was higher and CD4+ cell response was less robust among older adults for both men and women when compared to younger PLWH, similarly to results described elsewhere [25]. Information on cause of death was unavailable, and it is likely that at least some of the increased mortality among older adults is due to increased background risk of death from natural aging processes. Additionally, differences in CD4+ cell count response may contribute to the higher mortality observed among older patients. Indeed, although older adults had slightly higher average CD4+ cell counts at ART initiation compared to adults 40–49 years and adults 25–39 years, older adults experienced significantly lower CD4+ cell count response than younger patients after ART initiation. Studies have shown discrepant findings, with some showing similar results to ours [25], [32], and others finding comparable CD4+ cell count responses in older and younger adults [33], [34]. However, with the exception of the study by Grieg et al [25], all of the latter studies were conducted in resource-rich countries where adults may have a different baseline clinical profile than that of our population. Our findings on reduced CD4+ cell response after ART initiation among older adults are similar to those noted by Grieg et al [25] by demonstrating that this decreased response remains up to 36 months after ART initiation, and that the decreased response remained even after adjustment for factors potentially associated with differential CD4+ cell response post ART initiation.

Our study has a number of strengths. To our knowledge, it is the largest multi-country study investigating characteristics and outcomes of older PLWH enrolling in HIV care in sub-Saharan Africa. The data used in this study were derived from health facilities that are in many ways typical of HIV care settings in the region. Some limitations must be noted, however. Only 67% of patients had a CD4+ cell count at ART initiation and of these, only 67% had at least one post-ART CD4+ cell count. Patients missing CD4+ cell counts at ART initiation were similar to patients with CD4+ cell counts at ART initiation in facility location, year of ART initiation, point of entry, sex, and WHO stage at ART initiation. However, patients initiating ART in Rwanda were significantly more likely to have ART initiation CD4+ cell counts (86%) compared to patients enrolling in Kenya (72%), Mozambique (62%), and Tanzania (61%). While it is possible that individuals with ART initiation and follow-up CD4+ cell counts differ on CD4 response from those with missing ART initiation and/or follow-up CD4+ cell counts, it is unlikely that this difference would be substantially differential by age category, suggesting this is an unlikely explanation for the observed difference in CD4 response between older and younger adults. Further, our findings are similar to those of another multi-country analysis in Africa [25]. Additionally, we lacked information on co-morbidities which may increase with age, and thus were unable to explore whether they contributed to the higher death rates observed among patients aged ≥50 years of age. Lastly, a large proportion of patients were LTF, especially among younger adults. Whether these patients stopped treatment, transferred to another facility or died is unknown. Since some proportion of patients deemed LTF in our analyses are likely to be unrecorded deaths [35], [36], we expect that the true death rate in our population is likely higher than documented in our data.

## Conclusions

This analysis found that, across a large population including 199 health facilities in 4 sub-Saharan African countries, increasing proportions of adults newly initiating ART, and active in HIV care and on ART, are 50 years and older. These older adults are at increased risk of death, at lower risk of loss to follow-up and have less robust CD4+ cell count response after initiation of ART. Given trends in increasing representation of older adults among the population seeking HIV care in sub-Saharan Africa, there is the need to better understand CD4+ cell count response and to identify programmatic features associated with enhanced outcomes of older patients on treatment.

## Supporting Information

Table S1 Complete results of regression analysis: change in CD4+ cell count after ART initiation.
(DOCX)
