# Supplementary material for: Characteristics and Outcomes among Older HIV-Positive Adults Enrolled in HIV Programs in Four Sub-Saharan African Countries
Source: PLoS One. 2014 Jul 30;9(7):e103864. doi: 10.1371/journal.pone.0103864 (PMC4116238; doi:10.1371/journal.pone.0103864)
Supplement: Table S1 — Complete results of regression analysis: change in CD4+ cell count after ART initiation. (DOCX) [file pone.0103864.s001.docx]

**Table S1. Complete results of regression analysis: change in CD4+ cell count after ART initiation**

Analysis Of GEE Parameter Estimates

Empirical Standard Error Estimates

Standard 95% Confidence

Parameter Estimate Error Limits Z Pr > |Z|

Intercept 24874.42 1344.198 22239.84 27509.00 18.51 <.0001

CD4_lag_time 0.0039 0.0036 -0.0032 0.0111 1.09 0.2776

ART_cd4 -0.3028 0.0057 -0.3140 -0.2915 -52.66 <.0001

sex 0 21.8582 0.6099 20.6628 23.0535 35.84 <.0001

a_type 1 4.7376 1.9638 0.8886 8.5865 2.41 0.0158

a_type 2 10.9888 1.9012 7.2625 14.7150 5.78 <.0001

a_type 3 -0.5762 2.3333 -5.1493 3.9969 -0.25 0.8049

a_type 4 11.5041 2.3861 6.8275 16.1807 4.82 <.0001

a_loc 1 -13.3228 1.4139 -16.0939 -10.5517 -9.42 <.0001

a_loc 2 4.2571 1.3422 1.6264 6.8878 3.17 0.0015

CountryID 1 31.5621 1.3794 28.8586 34.2656 22.88 <.0001

CountryID 2 -24.7347 1.7576 -28.1796 -21.2898 -14.07 <.0001

CountryID 3 23.5652 1.5963 20.4366 26.6938 14.76 <.0001

art_year -12.2798 0.6687 -13.5905 -10.9691 -18.36 <.0001

age_group_artn 15-24 yrs 5.4836 2.5804 0.4260 10.5411 2.13 0.0336

age_group_artn 40-49 -7.9501 1.3797 -10.6543 -5.2459 -5.76 <.0001

age_group_artn 50 yrs and above -20.5947 1.7896 -24.1023 -17.0872 -11.51 <.0001

Key:

CD4_lag_time = time (days) between ART initiation and follow-up CD4+ cell count

ART_CD4 = CD4+ cell count (cells/mm3) at ART initiation

Sex: 0 = female, 1 = male (reference)

A_type = facility type, 1 = public primary, 2 = public secondary, 3 = public tertiary, 4 = private, 5 = other

A_loc = facility location (1 = urban, 2 = peri-urban, 3 = rural (reference)

Countryid = Country identifier (1 = Rwanda, 2 = Mozambique, 3 = Tanzania, 4 = Kenya (reference)

ART_year = year of ART initiation

Age_group_artn = age at ART initiation (15-24 yrs, 25-39 yrs (reference), 40-49 yrs, 50+ yrs)

CD4_diff (dependent variable) = difference between follow-up CD4 count and CD4 at ART initiation
